# Factors that act as facilitators and barriers to nurse leaders’ participation in health policy development

**DOI:** 10.1186/1472-6955-13-20

**Published:** 2014-07-10

**Authors:** Nilufa Shariff

**Affiliations:** 1 Advanced Nursing Studies Programme - Kenya, Aga Khan University, 3rd Parklands Avenue, Nairobi 00100, Kenya

## Abstract

**Background:**

Health policies impact on nursing profession and health care. Nurses' involvement in health policy development ensures that health care is safe, of a high quality, accessible and affordable. Numerous factors influence nurse leaders' ability to be politically active in influencing health policy development. These factors can be facilitators or barriers to their participation. There is scant research evidence from Eastern African region that draws attention to this topic. This paper reports part of the larger study. The objectives reported in this paper were those aimed to: build consensus on factors that act as facilitators and barriers to nurse leaders' participation in health policy development in Kenya, Uganda and Tanzania.

**Methods:**

A Delphi survey was applied which included: expert panelists, iterative rounds, statistical analysis, and consensus building. The expert panelists were purposively selected and included national nurse leaders in leadership positions in East Africa. Data collection was done, in three iterative rounds, and utilized a questionnaire with open and closed ended questions. 78 expert panelists were invited to participate in the study; the response rate was 47% of these 64.8% participated in the second round and of those 100% participated in the third round. Data analysis was done by examining the data for the most commonly occurring categories for the open ended questions and descriptive statistics for structured questions.

**Results:**

The findings of the study indicate that both facilitators and barriers exist. The former include: being involved in health policy development, having knowledge and skills, enhancing the image of nursing and enabling structures and processes. The latter include: lack of involvement, negative image of nursing and structures and processes which exclude them.

**Conclusion:**

There is a window of opportunity to enhance national nurse leaders' participation in health policy development. Nurse leaders have a key role in mentoring, supporting and developing future nurse policy makers.

## Background

Nurses constitute the largest health care workforce in most countries. An estimated 35 million nurses make up the greater part of the global health workforce [1]. Nurses interact closely with patients and their families and often accompany patients around the clock in all sectors of health care. This gives nurses a broad appreciation of health needs, of how factors in the environment affect the health situation for clients, their families and communities and of how people respond to different strategies and services. Nurses command expert knowledge based on their education and experience that could contribute positively towards improving all spheres of health care. ICN [2] reiterates that nurses can make a major contribution in promoting and shaping effective health policy because they closely interact with clients, gaining an appreciation of the health needs of the population and factors that influence these health needs.

The purpose of this study was to build consensus among nurse leaders’ on factors that facilitate or deter their participation in health policy development from the East African context. Policy in the context of this paper refers to the principles that govern a chosen course of action or inaction towards attainment of goals which influence the interest of the public [3]. Health policies are guidelines, directives or principles pertaining to the health sector that govern the action or inaction that influence the health of the population [4].

### International context on nurses’ participation in health policy

National health policies impact on nursing profession and health care. Studies reviewed from USA, Australia, New Zealand, and Canada, revealed that national health policy reforms were often related to budget cuts. These reforms resulted in downsizing nurse staffing, and, in turn, created ripple effects on nurses and patient care. The impact largely took the form of negative consequences for nurses and patients in terms of: decreased staffing, increased workload, decreased job satisfaction, job insecurity and decreased quality and quantity of patient care, growth in numbers of unlicensed personnel, and ethical dilemmas [5-8]. Some positive effects, such as nurses becoming more assertive and gaining autonomy, ensued [9].

Nurses demonstrate some degree of political participation, although their level of political participation is restricted. Politics in this context is defined as striving to share power or to influence the distribution of power among groups within the state [10]. Political activity refers to being part of groups and participating in activity to influence health policy. In studies undertaken in the USA [9-12], results indicated that most nurses participated in political activities and thought that people like them could influence government activities. Conversely, Chan and Cheng’s [13] study in Hong Kong found that political activism was insufficient and disagreed that nurses had the power to influence government policy. Furthermore, a more recent study conducted by Kunaviktikul et al. [14], explored involvement of nurses, those in hospital based clinical nurses and national nurse leaders, in health policy in Thailand. Reported findings indicated that the majority of the former were not involved in national health policy development. Nurse leaders’ were not involved in policy formulation or modification stages but were involved in the policy implementation stage. These findings suggest that whilst nurses are not apolitical, they appear to be at various levels of political engagement in different countries. There is no clear consensus on nurses’ political efficacy in relation to whether or not they believe they are able to influence government health policy.

Numerous factors influence nurses’ ability to be politically active in influencing health policy development, such as finding needed time and possessing relevant knowledge and interest about how political issues affect health care and the nursing profession [11-15]. Historically, nursing has suffered from a poor public image that it has found difficult to cast off [16-18]. For example, Florence Nightingale’s vision and influence at the national health policy level completely changed society’s views of nursing [18]. Nursing, however, has not since been able to sustain this influence at policy level, and the image of nursing has remained low compared to other professions such as medicine. Moreover, medical technological advances and medical and curative dominance within health care has moved nurses’ focus from preventive and promotive care to individual care and cure. This has resulted in significant withdrawal of nursing from social and political activism and in the lessening of the reputation of nurses as a social change agent; as a result, there has been a loss in nursing power as regards policy making [19]. Although the need for political action and policy influence has been recognized, nursing education has been slow to respond to this call [20]. The preparation imparted by nursing education, does not adequately equip nurses with the knowledge and skills necessary for involvement in policy development [21-23]).

Studies reviewed reveal that it is possible for contemporary nurses to influence health policy. Certain factors can enhance nurses’ ability to participate in the policy development process. Factors include: being involved and gaining experience in policy development, having role models, being educated and knowledgeable about health systems and policy development process, political activism, conducting research to expand knowledge, being supported by professional organizations and developing leadership skills [13,24-27]. When nurses are involved and successfully influence health policy development, there are clear benefits to the patient, the profession and the nurse.

Nurses’ involvement in health policy development ensures that health care is safe, of a high quality, accessible and affordable [28]. Nurses have made progress in political participation and they exert influence in health policy development in some industrialized countries such as the United States of America (USA) and the United Kingdom (UK). In studies conducted by Latter and Courtenay (2004), While and Biggs (2004) and Bradley and Nolan (2007) in the USA and UK indicate that nurses’ involvement in health policy development and reform positively influences health care. These studies indicated that when nurses participated in policy development they were able to make valuable contributions and positively influence areas that include: access to health services; suicide prevention in adolescents; development of guidelines for the care of pregnant women and their children; child abuse policy; authorizing nurses to prescribe medication within the framework of the Nurse Prescribers Formulary (NPF); and improving continence services. Whilst there is a gap in literature with regards to the impact of these policies on patient care some literature from the UK with regards to Nurse prescribing indicates that: patients were generally satisfied, received quicker treatment, nurses’ experienced enhanced job satisfaction, nurses perceived enhanced quality of their practice, and skills, enhanced nurse self-esteem, enhanced autonomy and enhanced professional role [28-30]. These studies indicate that nurses’ influence on policy can have a positive impact in this regard.

### African context on nurse participation in health policy

In the African context, a woman’s life is complicated by with environmental, socioeconomic and psychological factors such as poverty, illiteracy, laborious domestic work, disease and abuse that are debilitating and unfair [31]. Confounding these circumstances is the traditional patriarchal system in which the decision-makers are men in the household. These factors and others contribute to the comparatively lower status of women in East Africa. The patriarchal social order also is reflected in the overall health care system [32]; Health care is not gender neutral. In a study that was conducted in South Africa by Van Der Merwe [33], the author concluded that, “Nurses as women within the traditional African setting at home were considered to be different, separate and not equal to men”. Patriarchal forces extensively affect the nursing workforce, which is constituted mostly of women [34]. Gendered social ideals have significant implications for the roles, responsibilities, and capabilities of individuals [34]. Nurses and nursing image are inherently linked to the dynamics that affect women.

The majority of the nurses in the East African Region are educated at the diploma level as an entry point into the profession; this level of education renders them less educated than other health care professionals [35,36]. Nursing education mainly focuses on clinical skills and theory related to patient care and management, not on leadership development or policy issues [35,36]. Access to higher education at the tertiary level is limited and expensive.

Organizational structures within which the majority of nurses are positioned and have functioned, particularly in the East African context, are bureaucratic. Policies, power and decisions are vested in the top level managers of the organization, whereas, the lower levels are mainly involved in implementation of those decisions [26,37,38]. There are few nurses, despite their being the largest workforce in health care, represented in senior management; nurses in these positions often adopt the ethos of senior management and represent management values rather than nursing issues or values [39]. Interestingly, there also is a higher proportion of male nurses in senior management positions compared to the proportion of males in the profession [40]. Workplace conditions for nurses commonly discourage participation in policy development. In a significant African study conducted by Phaladze [41] who investigated the role of nurses in the Human Immunodeficiency Virus/Acquired Immune Deficiency Syndrome (HIV/AIDS) policy process in Botswana, concluded that the majority of the nurses were aware of the national HIV/AIDS policy. A small minority, however, were involved in the policy development process, mainly in the policy adoption, implementation and evaluation stages. The reasons for non-involvement included lack of confidence in nurses’ ability to competently participate in policy decisions and lack of being proactive towards issues related to HIV/AIDs. This situation is evident in the Kenyan context where a study conducted by Evans and Ndirangu [42] found that nurses were excluded from HIV/AIDs health policy development, decisions were made in a top down fashion, and nursing involvement was limited to implementing health policies.

In summary, review of the literature reveals that there are interrelated and complex factors that influence and contribute to limited nurses’ participation in health policy development. Nurses face challenges in being involved in health policy development at the grassroots level, as well as at the government level. Nurses believe that they are excluded and are not part of the health policy development process and that they are not present in large enough numbers to make a difference. Other major factors acting as barriers to participation include inadequate political and policy development skills, lack of status of women that also shapers the image of nursing, lack of education and lack of supportive organizational structures. However, research studies reveal that when nurses are involved and successfully influence health policy development, there are benefits to health care delivery. There are factors which could facilitate nurses’ participation in this regard include: effective leadership, political savvy, education, knowledge and understanding the health policy development process.

Whilst there is some literature from the developed world examining factors that influence nurses’ and their leaders’ participation in health policy development, there is limited literature from the developing world, particularly from Africa, and specifically the East African context. This paper reports ***part*** of a larger study that aimed to: “build consensus on factors that act as facilitators to nurse leaders’ participation in health policy development in East Africa” and “build consensus on factors that act as barriers to nurse leaders’ participation in health policy development in East Africa”.

## Methods

### Research design

A Delphi survey was applied and included the following: expert panelists, iterative rounds, statistical analysis, and consensus building [43]. The study was conducted in the three East African countries of Kenya, Uganda and Tanzania.

### Sampling framework

The target population for this study comprised of nurse leaders who occupied national or provincial leadership positions in East Africa. The sample was derived from the target population and consisted of nurse leaders working in national or provincial leadership positions from the Ministry of Health (or equivalent), Nursing Councils, National Nurses Associations and Universities. Purposive sampling was used because the intention was to include participants who were knowledgeable about the subject being studied. The sampling framework developed for the study required that the sample meet the following criteria: (1) Registered Nurse; (2) employed in senior nursing leadership/management capacity in East Africa, and (3) currently working at the national/provincial (regional) level or at a university.

Additionally, the “closeness continuum” developed by Needham and de Loë ([44];138) was applied. As per the criteria proposed in the “closeness continuum”, nurse leaders with subjective expertise, mandated expertise and objective expertise were included in the study. Subjective expertise included possessing knowledge derived from experience in health policy implementation, from having been affected by health policy, or from having participated in the health policy development process. For the purpose of this study, participants included Provincial Nurses/Public Health Nurses and leaders in the National Nurses’ Professional Associations who may also have worked as registered nurses. Mandated expertise entailed knowledge and experience in terms of the job requirement related to participation in the health policy development process. For the purpose of this study, participants included Chief Nurses/ Registrars/ Chairpersons of Nursing Regulatory Bodies/ National Nurses’ Professional Associations Leaders/Deputies. Objective expertise entailed knowledge gained through academic position, education and research with regards to the policy development process. For the purpose of this study, participants included Academic Nurse Leaders/Deans/Academic Heads. The study provided an opportunity for 78 nurse leaders from the three East African countries to be part of the study, which was conducted in three iterative rounds: 37 (47%) responded in the first round. Of the 37 expert panelists invited to participate in the second round, 24 (64.8%) participated: all 24 (100%) participated in the third round.

### Questionnaire development

The data collection tool was developed by the researchers. The tool was a questionnaire and was developed with reference to research literature. The Round 1 questionnaire aimed to collect qualitative data and included two sections. *Section 1* covered demographic data on the panelists with reference to country represented, organization represented, number of years of experience in nursing, and number of years in current position. The demographic data helped to confirm that the sample was representative of nurse leaders as proposed in the sampling framework and that participants possessed the critical characteristics relevant to achieving the aim of the study. *Section 2* aimed to “build consensus on factors that act as facilitators to nurse leaders’ participation in health policy development in East Africa” and “build consensus on factors that act as barriers to nurse leaders’ participation in health policy development in East Africa”. Round 2 and 3 questionnaires were a reflection of the data collected in Round 1 and were developed into quantitative questionnaires.

Pre-testing of the questionnaires was conducted, in all rounds. The participants who were selected to pretest the tool comprised a purposive sample of nurse leaders (senior management level positions whose role included participation in the policy development process). These individuals were excluded from the main study. The participants indicated that they found: the questions clear; language acceptable; topic relevant to nursing and related to health policy development; and the instrument user friendly.

### Validity

Delphi surveys mainly are concerned with face validity and content validity. *Face validity* was achieved by pre-testing the tool for exhibiting: clarity of content, being reflective of the topic studied, clarity of language, being unambiguous and readable [45]. *Content validity* was enhanced in three respects. First, questionnaires were developed with reference to the literature related to health policy development and nurses’ participation in health policy development [46]. Second, all three questionnaires were pre-tested with a representative sample of nurse leaders, to ensure that the concepts included in the study were actually related to health policy development process. Third, the purposive study sample was comprised of a panel of experts who participate in the health policy development process.

### Data analysis

The qualitative data from the open ended questions in the Round 1 questionnaire were transcribed verbatim into Word documents; the documents were read for relationships and patterns. Similarities and differences were identified; words and phrases were grouped by cutting and pasting the Word document into clusters of similar ideas and concepts and by highlighting in different colours. This procedure aided in grouping similar ideas together and identifying the most commonly occurring attributes and concepts. The analysis of this phase was undertaken independently by the researcher and an assistant; individual notes were compared to validate the concepts that occurred. The concepts that most commonly occurred were then developed by the researcher into questions for questionnaire. The areas that deferred were noted and reevaluated by the researcher and assistant.

For the quantitative (Rounds 2 and 3) data analysis the computer package Statistical Package for Social Scientists, version 15, (SPSS 15) was utilized. These questionnaires utilized a Likert scale with numerical values attached to the scale range: one (1) indicated strongly agree and five (5) indicated strongly disagree. Data were analyzed utilizing descriptive statistics. Data reported in this paper are mainly frequency distributions that include summaries of categories, percentage agreement, mean and standard deviation.

### Consensus

In this study, consensus was built over three rounds. The first round generated unstructured data that are presented in the first column as labeled in the tables. The second and third rounds gave the expert panelists an opportunity to reevaluate their ideas (consensus building) in line with the group’s ideas that were presented in summaries and descriptive statistics.

The parameters set for Round 2 were percentage agreement of ≥90%, and for Round 3 were ≥70%, a mean value of <2 and standard deviation of <2; these were regarded as convergence of opinion towards agreement and consensus for the purpose of this study. Conversely, a percentage agreement of <90% (2^nd^ round) or <70% (3^rd^ round), mean of >2 and standard deviation of >2 was considered as divergence of opinion. Categories that did not achieve convergence in the second round were omitted from the third round; these have been left blank and highlighted in the tables.

### Ethical considerations

Ethical approval was obtained from the Health Studies Research and Ethics Committee of the College of Human Sciences, University of South Africa (reference number - project number 3273 404 2). Approvals were secured from the National Councils concerned with research clearance in Kenya, Uganda and Tanzania. Right to autonomy was respected and informed consent attained by explaining the benefits, rights and risks involved in the research study in writing. Consent to participate was assumed by the return of the questionnaire. Confidentiality was maintained at all times throughout the study’s three phases of collection. Once the questionnaires were returned they were stored securely. The participant’s mail addresses, required for subsequent rounds, was delinked from the questionnaire. The information collected was presented anonymously as group views. This meant that the data presented in aggregate form, representing the collective views of the expert panel members [47].

### Data collection process

A database of expert panelists was created through networking with relevant offices because a database with the current information pertaining to the nurse leaders was unavailable. This database formed the basis for developing the sampling framework. The questionnaires were delivered to all the 78 nurse leaders incorporated in the study via email, and printed copies were hand delivered and also sent to their postal addresses with self-addressed envelopes to facilitate return of the questionnaire. Follow up reminders were made via email and by telephone. The data collection process was conducted between September 2009 and May 2010, for the three rounds.

## Results

### Demographic data

The demographic data indicated that the participants were representative of the target population identified as having had opportunity to be part of the policy making arena. A majority, 16 (43.2%) of the participants were from Kenya, with 15 (40.6%) from Tanzania and 6 (16.2%) Uganda. Participants were mainly from urban settings. A majority of the participants were from the Ministry of Health (17; 45.9%) or academic organizations (10; 27%). Most of the participants were more than 40 years of age (89%) and females (62.2%). More than eighty percent of the expert panel members, 31 (86%), reported more than 15 years of experience in nursing, indicating that they had considerable experience and expertise in the nursing profession. Almost three quarters, 27 (73%), of the expert panelists reported up to five (5) years of experience in their current position. Whilst the majority of the expert panelists 31 (86%) reported more than 15 years of experience in nursing, only over a quarter (27%) had 6 to 15 years of experience in their current policy related position. This demographic may suggest that nurse leaders do not remain in health policy positions for long periods and that by the time they secure policy related positions they are older and nearer retirement. As such, nursing might be losing nurse leaders with experience and expertise in health policy to retirement.

The concepts identified are shown in the first column of the tables. The second and third rounds built consensus on the most critical and important elements of these concepts and are shown in the second and third columns of the tables. A high degree of consensus was achieved about facilitators identified to enhance participation in health policy activity.

### Factors that act as facilitators to nurse leaders’ participation in health policy development in East Africa

The analysis of the data revealed that there were factors that acted as facilitators to nurse leaders’ participation in health policy activity. These facilitators included: being involved; being knowledgeable and skilled, being supported; positive image of nursing; enabling structures and available resources.

**Being involved** was identified as a facilitator of nurse leaders’ participation in health policy development in Round 1. Involvement included having experience and exposure; being accorded opportunity; being present at all stages of policy development; seeking opportunity for participation and being active participants (see Table 1). There was consensus in Rounds 2 and 3 among the expert panelists on the facilitators related to involvement in the policy development process.

**Table 1:** Being involved

**Round 1 (n = 37)**	**Round 2 (n = 24)**			**Round 3 (n = 24)**		
	**PA**	**M**	**SD**	**PA**	**M**	**SD**
Nurse leaders must have experience and exposure to health policy development process	96%	1.67	0.76	96%	1.21	0.51
Nurse leaders must have opportunities to participate in forums where policies are formulated by policy makers	100%	1.42	0.58	100%	1.14	0.35
Nurse leaders must have opportunities to be included by policy makers at every stage of the health policy development process	96%	1.29	0.86	100%	1.25	0.44
Nurse leaders must participate actively in the entire policy making process when given the opportunity to participate	100%	1.21	0.42	100%	1.23	0.43

**Being knowledgeable and skilled** were also identified as facilitators of nurse leaders’ participation in health policy development. These included being knowledgeable and skilled in health policy development; possessing a university education; and content related to health policy being covered in the curriculum (see Table 2). There was consensus in Rounds 2 and 3 that being knowledgeable and skilled were facilitators to nurse leaders’ participation in the policy development process.

**Table 2:** Being knowledgeable and skilled

**Round 1 (n = 37)**	**Round 2 (n = 24)**			**Round 3 (n = 24)**		
	**PA**	**M**	**SD**	**PA**	**M**	**SD**
Nurse leaders must be knowledgeable and skilled in the health policy development activities at all levels	96%	1.42	0.78	100%	1.17	0.39
Nurse leaders must have at least a university degree - level of education (BScN)	91%	1.63	0.97	91%	1.65	0.89
Content related to health policy development must be included in the basic nursing education	100%	1.54	0.66	91%	1.55	0.80

**Being supported** was identified as a facilitator of nurse leaders’ participation in health policy development. Being supported included benefiting from role models, supportive mentorship and networks for support and sharing experiences (see Table 3). There was consensus among the expert panelists that support was necessary to facilitate nurse leaders’ inclusion and involvement in the policy development activities in rounds 2 and 3.

**Table 3:** Being supported

**Round 1 (n = 37)**	**Round 2 (n = 24)**			**Round 3 (n = 24)**		
	**PA**	**M**	**SD**	**PA**	**M**	**SD**
Nurse leaders must have role models through whom they can learn to participate in the health policy development process e.g. directors of medical services who are involved in health policy development	96%	1.42	0.72	100%	1.50	0.51
Nurse leaders must receive supportive mentorship from leaders who have been involved in and have actively participated in health policy development	100%	1.33	0.49	100%	1.25	0.44
Nurse leaders need to have networks for support and to share experiences on policy related issues (e.g. national nurses’ association – intensive care nurses’ chapter)	100%	1.42	0.50	100%	1.33	0.34

**Positive image of nursing** was identified as another facilitator. This findings suggests that nursing must be considered a valuable partner in policy development, and nurses with potential must be appointed in policy making positions while they also must engage policy makers and engage the media to change the image of nursing (see Table 4). There was consensus among the expert panelists on the facilitators related to the image of nursing.

**Table 4:** Positive image of nursing

**Round 1 (n = 37)**	**Round 2 (n = 24)**			**Round 3 (n = 24)**		
	**PA**	**M**	**SD**	**PA**	**M**	**SD**
Nurse leaders’ input in policy development must be respected by policy makers	100%	1.38	0.50	100%	1.29	0.56
Nurse leaders with the ability (right credentials) to influence health policy should be nominated to national leadership positions e.g. Director of Nursing Services	100%	1.25	0.53	100%	1.17	0.38
Nurse leaders must engage policy makers to ensure a bottom up and top down approach during the entire policy development process	100%	1.54	0.72	100%	1.35	0.49
Nurse leaders must have the ability to engage the media to change the image of nursing	91%	1.71	0.91	100%	1.27	0.46

**Enabling structures** emerged as a facilitator of nurse leaders’ participation in health policy development in Round 1. Enabling structures included a legislature which ensures that national nurse leaders are included in policy development; a directorate of nursing services; enhancing the numbers of nurses at policy development level; nurses with ability in health policy activity; and a gender balance (see Table 5). There was consensus in Rounds 2 and 3, among the expert panelists on the structures that facilitate nurse leaders’ participation in the policy development process (see Table 5).

**Table 5:** Enabling structures

**Round 1 (n = 37)**	**Round 2 (n = 24)**			**Round 3 (n = 24)**		
	**PA**	**M**	**SD**	**PA**	**M**	**SD**
A legislature which ensures that national nurse leaders are included in the health policy development process	100%	1.38	0.65	100%	1.29	0.46
Nursing must have a director of nursing services who is on a par with the director of medical services (or equivalent) at the ministry of health or equivalent	100%	1.42	0.72	92%	1.38	0.65
Leadership positions must be allocated for nurse leaders at policy making levels (affirmative action)	100%	1.46	0.66	92%	1.29	0.62
Policy makers must enhance the representation (numbers) of nurse leaders at national policy making level	96%	1.75	2.03	100%	1.26	0.45
Policy makers must ensure that they have a gender balance (nurse leaders must be proportionate to the percentage of women and men in the nursing profession) at health policy development positions	90%	2.13	0.90	74%	1.83	1.03

**Available resources** the availability of resources also emerged as a facilitator for nurse leaders’ participation in health policy development. This included having resources; having business and financial skills; and being able to mobilize them for policy making activity (see Table 6). There was consensus in (rounds 2 and 3) among the expert panelists that resources are required for participation in health policy development activities.

**Table 6:** Available resources

**Round 1 (n = 37)**	**Round 2 (n =24)**			**Round 3 (n =24)**		
	**PA**	**M**	**SD**	**PA**	**M**	**SD**
Nurse leaders must have resources allocated for their participation in policy development activities e.g. financial, material and human	91%	1.58	0.93	100%	1.17	0.39
Nurse leaders must be able to mobilise funds to finance policy making activities	96%	1.79	0.72	92%	1.63	0.65

### Factors that act as barriers to nurse Leaders’ participation in health policy development in east Africa

Reported here are the factors that act as barriers and hinder nurse leaders’ participation in health policy development. The categories identified in the first round were: lack of involvement; lack of knowledge and skills; negative image of nursing; lack of enabling structures and lack of resources. There were a number of areas that were identified as barriers in Round 1, however, that did not achieve consensus in Round 2. These areas were omitted from Round 3 as indicated by the blank areas in the tables.

**Lack of involvement** emerged as a barrier to nurse leaders’ participation in the health policy development process in Round 1. Lack of involvement included: input called upon on an ad hoc basis; lack of opportunity; lack of opportunity at various levels; lack of forums; lack of experience; lack of active participation; top down approach to policy making and poor planning on the process of problem identification (see Table 7). There was consensus (Rounds 2 and 3) among the expert panelists on two barriers related to lack of opportunity to become involved in health policy development. The remainder of the barriers related to lack of involvement did not attain strong consensus as required for this study and were omitted from Round 3.

**Table 7:** Lack of involvement

**Round 1 (n = 37)**	**Round 2 (n =24)**			**Round 3 (n =24)**		
	**PA**	**M**	**SD**	**PA**	**M**	**SD**
Nurse leaders’ input is called upon on an ad hoc basis and they are not part of the full policy process	100%	1.54	0.77	88%	1.71	0.91
Lack of opportunity for nurse leaders to be involved in the whole process of policy development	100%	1.58	0.72	91%	1.61	0.78
They lack forums to discuss policy problems and agenda items within nursing at national level	80%	2.33	1.20			
Lack of experience necessary for active participation in the health policy development process	74%	2.33	1.40			
Inability to actively participate in the policy process when given the opportunity	64%	2.67	1.37			
There is poor planning by the nurse leaders on the process of problem identification and agenda setting	53%	2.83	1.37			

**Lack of knowledge, skills and support** emerged as barriers to nurse leaders’ participation in policy activity. These included: lack of tertiary education, lack of knowledge pertaining to health policy development process and throughout the stages of policy development, as well as lack of support and confidence (see Table 8). In Round 2, although two of the barriers were close to the convergence threshold of 90%, none met this criteria for consensus among the expert panelists on all barriers related to knowledge and skills for participation in the policy development process. This indicated a divergence in opinions that was interpreted as disagreement and lack of consensus. These barriers were therefore omitted from the next round.

**Table 8:** Lack of knowledge, skills and support

**Round 1 (n = 37)**	**Round 2 (n =24)**			**Round 3 (n = 24)**		
	**PA**	**M**	**SD**	**PA**	**M**	**SD**
Their level of education is low, that is, they lack a university level of education	48%	3.21	1.38			
Lack of relevant knowledge and skills necessary to participate in the policy development process	59%	2.71	1.49			
They lack knowledge and skills relevant to problem identification and agenda setting	63%	2.63	1.53			
Lack of knowledge of the health policy formulation guidelines	71%	2.50	1.25			
Lack of a clear understanding of the health policy implementation process	74%	2.21	1.29			
Policies being unclear to the nurse leaders who are expected to implement them	88%	1.92	1.06			
Lack of knowledge and skills of the policy evaluation process	82%	1.13	1.36			
They lack a supportive environment in terms of mentorship and encouragement	88%	1.79	0.98			
They lack information about the policy development forums	82%	2.25	1.07			
Lack of confidence to air their views, related to policy issues, to the policy makers	65%	2.63	1.28			

**Negative image of nursing** emerged as a barrier and included: nurses’ contribution to the policy process not being recognized and lack of opportunity to be involved in health policy development. There was consensus (Rounds 2 and 3) among the expert panelists on the barriers related to the image of nursing and its effect on nurse leaders’ participation in the development of health policy (Table 9).

**Table 9:** Negative image of nursing

**Round 1 (n = 37)**	**Round 2 (n =24)**			**Round 3 (n =24)**		
	**PA**	**M**	**SD**	**PA**	**M**	**SD**
Nurse leaders’ potential contribution to the policy process is not recognized as significant by the policy makers	96%	1.65	0.78	83%	1.88	0.90
Nurse leaders’ lack of opportunity to be involved in the policy development process by the policy makers	100%	1.17	0.38	70%	1.96	1.11

**Lack of enabling structures** was identified as a barrier to nurse leaders’ participation in health policy and included: institutional structures and systems exclude nurse leaders; health policies are developed at national level and then rolled down to other levels; inadequate representation of nurse leaders; policy making positions are given to doctors; and other health professionals including doctors represent nurses and nursing issues at health policy development forums (see Table 10). There was consensus (Rounds 2 and 3) among the expert panelists on all except two of the structural barriers to nurse leaders’ participation in the policy development process as indicated in Table 10. This study include a higher number of males compared to the national average which might have influenced this percentage average.

**Table 10:** Lack of enabling structures

**Round 1 (n = 37)**	**Round 2 (n = 24)**			**Round 3 (n = 24)**		
	**PA**	**M**	**SD**	**PA**	**M**	**SD**
Institutional structures and systems are such that they exclude nurse leaders from being part of the policy process e.g. nurse leaders are in relatively junior positions	96%	1.58	0.83	75%	2.04	1.12
Health policies are developed at national level and then rolled down to other levels (district, provincial & national) for implementation	100%	1.46	0.72	91%	1.55	0.80
Inadequate representation (numbers) of nurse leaders at the policy making forums	96%	1.54	0.83	92%	1.54	0.66
Most appointments into policy making positions are given to doctors	100%	1.29	0.55	100%	1.13	0.34
Other health professionals including doctors represent nurses and nursing issues at health policy development forums as structures are not inclusive of nurse leaders	100%	1.58	0.78	88%	1.54	1.02
Most policy making positions are given to male leaders; thus female leaders cannot participate (gender imbalance)	67%	2.63	1.14			
Most of the nursing leadership representatives at health policy development level are as a result of political appointments	68%	2.46	1.38			

**Lack of available resources e**merged as a barrier for nurse leaders’ participation in health policy development. These include not possessing resources and being able to mobilize these for policy making activity. Influencing policy development and the course of the health policy is largely about securing resources for health care, work which in itself requires resources (see Table 11). There was divergence of views in the second sub-concept as indicated in Table 11.

**Table 11:** Lack of available resources

**Round 1 (n = 37)**	**Round 2 (n = 24)**			**Round 3 (n = 24)**		
	**PA**	**M**	**SD**	**PA**	**M**	**SD**
Lack of financial, material and human resources to implement health policy	91%	1.75	1.07	83%	1.83	
Lack of funds and resources to attend the forums at which the policies are developed	68%	2.63	1.31			

## Discussion

The aim of the study was to build consensus on factors that act as barriers or facilitators to Nurse Leaders’ participation in health policy development. Key factors were identified from the analysis that were considered to be facilitators and barriers to nurse leaders’ participation in health policy activity. Facilitators included: being involved; possessing knowledge and skills; being supported; positive image of nursing; enabling structures; and having resources. Barriers included: lack of involvement; negative image of nursing; lack of structures; and resources. The facilitators and barriers were largely interconnected. The concept of support was a facilitator but did not appear to be a strong barrier. All factors identified as facilitators to nurse leaders’ participation in health policy in Round 1 attained a high degree of consensus in Rounds 2 and 3 and were retained. Whilst most concepts identified as barriers to participation attained consensus in Rounds 2 and 3, there were some barriers that did not attain consensus in Round, and were omitted from Round 3 of the study.

### Facilitators to nurse Leaders’ participation in health policy development

The findings indicate that *knowledge and skills* are an important precursor to nurse leaders’ participation in health policy activities. nurse leaders’ participation in health policy development can be enhanced if they have attained knowledge and skills in health policy development activities. These findings concur with Rains and Carroll [48] findings that indicated that being educated on health policy led to increased self-perceived competence in knowledge, skills, and understanding within the context of health policy activity. Furthermore studies by Byrd et al. [49], Primomo [38], Primomo and Bjorling [39] indicate that exposing students to learning related to policy and knowledge of the legislative and policy processes enhances their political involvement. Furthermore, knowledge and skills can be enhanced if nurse Leaders enjoy access to supportive mentorship and role modeling from leaders who have been actively involved in policy development activities. Sundquist’s [50] study similarly found that support, encouragement and inspiration were necessary for participation in health policy development. Noteworthy is Dollinger’s [25], study that indicates that even well-educated nurses who work within the government in the USA find it a challenge to influence health policy. However it is not clear whether these nurses had learned about policy development and issues pertaining to political activity. The current study extends current knowledge, derived from studies in other contexts, and provides evidence that this facilitator is relevant in the East African context.

The results also indicate that *being involved* in policy development activity was a facilitator for participation in policy development. Participation in policy development can be enhanced if nurse leaders’ are accorded opportunities to be involved in the health policy development process. This suggests that enhancing nurse leaders’ involvement in policy development activity could enrich their experience, confidence and competence in the process. A recent study by Taylor, Fair and Nikodem [51], conducted in South Africa, with health professionals to examine their suggestions towards improving HIV-related maternal care, confirmed that nurses would like to be involved in health policy decision making. Other studies also are consistent with current study findings [13,26,37]. Nonetheless, nurse leaders’ must be proactive in seeking opportunity to be involved through various forums including those supported by their National Nurses Associations. This may enhance nurse leaders’ political influence through factors such as power of numbers, unity and collective voice. Furthermore, current study findings support the importance of providing nurse leaders with opportunity to gain experience and competence in participating effectively in policy development to having their voices heard and honored.

The findings of the current study indicate that a positive image of nursing facilitates Nurses’ participation in policy activity. Farsi, Dehghan-Nayeri, Negarandeh and Broom, recommend that to alter nurses’ social position, they must be involved in policy-making and political affairs [52]. A positive public image of nursing dictates how society will value nurses and their input in health policy decision making and further will dictate whether or not nurses will actually be part of the process.

Furthermore, the findings of the current study suggest that enabling structures can enhance and position nurse leaders’ effectively in health policy forums and activities. Similarly, other studies indicate that nurses are not able to influence health policy development as they are not present in large enough numbers and therefore need to increase their numbers in policy activities [24,53]. Influencing policy development and the course of the health policy is largely about securing resources for health care; and the work of participating in policy development activity also is in itself resource intensive. Small’s [54] and Casey’s [55] findings indicate that a correlation between income and political behaviour to influence policy: as income increases, so does political participation. As Nurses gain confidence and experience in policy development activities they may be able to influence institutional structures and resources that favour nurses’ inclusion and participation in these activities.

### Barriers to nurse leaders’ participation in health policy development

The study revealed a number of barriers to nurse leaders’ participation in health policy activities. These include: lack of involvement; negative image of nursing; lack of structures; and resources. However, not all barriers identified in Round 1 were retained by the expert panelists as the iterative rounds progressed and as the expert panelists re-evaluated their ideas in relation to the group summaries and statistics. This indicated that the study objectives of building consensus were met and that the expert panelists reviewed and re-evaluated their ideas in relation to other panelists’ ideas. Elimination of certain barriers suggests that the barriers were not altogether applicable to the entire group of expert panelists. Furthermore, this study set a high level of consensus as the threshold for convergence. The barriers that did not attain universally strong consensus were all barriers related to knowledge and skills, some barriers related to involvement, and a few barriers related to structures and resources.

The findings of the current study indicate that whilst the expert panelists believe that knowledge, skills, and education are necessary for participation in health policy, lack of these are not a barrier to their participation. Significantly, majority of expert panelists in this study (70%) held a basic degree. In contrast to current study findings, Kunaviktikul et al’s [14] study indicated that lack of knowledge and skills related to health policy was a barrier for nurse leaders’ participation in policy activity in that study. Furthermore, the current study findings indicate that nurse leaders’ are excluded from the health policy development process. They lack opportunity to be part of the whole process; instead, they tend to be called upon on an ad hoc basis, which results in an inadequate representation and preparation for and hence in participation in policy forums. Opportunity to be involved would engender experience, confidence and competence; hence, lack of opportunity may lead to deficiency in involvement. However, there was not adequate consensus in this study with regards to Nurses’ participation being hindered by: lack of forums, lack of experience, inability to actively participate and planning by nurse leaders.

Furthermore the findings indicate that a negative public image of nursing appears to hinder nurse leaders’ ability to be part of the policy making arena. The negative public image of nursing undermines nurse leaders’ inclusion in the health policy development process. Their potential contribution to this process is not recognized as significant and they are not included in it by policy makers. These findings are supported by Phaladze’s work [41] where she examined the role of nurses in HIV/AIDs policy development in Botswana and concluded that the reasons for nurses being excluded from the policy development process included the negative image of the profession amongst policy makers. Furthermore, Dollinger [25] concluded that nurses in the USA, exhibited little ability to influence policy due to the lack of status of the nursing profession and the dominance of the medical profession in government.

In addition, structures exclude national nurse leaders from the policy development process. These structures confine nurse leaders to occupying relatively junior positions, recruitment policy for national nurse leadership being unclear and health policies are developed at national level. The majority of policy development appointments are given to doctors and other health professionals who then represent nursing issues at the health policy development forums. The top-down approach toward policy development is not novel: numerous authors cite this problem [41,54,55] and all cite top down management at government level, with the government setting the priorities and nurses being the implementers. Opportunity to be involved would engender experience; hence, lack of opportunity leads to a deficiency of experience. There was divergence of opinion with regards to most policy making positions are given to male leaders and nursing leadership positions are political positions. Of interest is that in this study did include a higher number of males (37.8) and this may have influenced this perspective.

### Push and pull factors that either enhance or deter participation in health policy development

These barriers, whilst fewer than the facilitators, appear to be formidable as they exclude a significant portion of these leaders from participation in the policy development process. Further, these barriers could potentially deprive Nurse Leaders’ of the opportunity to gain knowledge, experience, and expertise and thus impeding their participation in the health policy development process. These facilitators (f) and barriers (b) were interconnected and there seem to be push and pull factors that included: being involved (f) and lack of involvement (b), being knowledgeable and skilled (f) and lack of knowledge and skills (b), enabling structures (f) and lack of enabling structures (b), positive image of nursing (f) and negative image of nursing (b), and availability of resources (f) and lack of resources (b). Although the concept of support was a facilitator but did not appear to be a strong barrier.

A spiral effect appears to be created whereby nurses’ lack of involvement in the process results in their limited knowledge and experience, potentially resulting in lack of confidence and further minimal input if they are invited to participate which may lead to being left out or not involved in any meaningful way. This spiral further prevents nurses from gaining experience and exposure and being involved in policy processes. There is ample opportunity to enhance nurse leaders’ participation in health policy development since there are more facilitators then barriers in this respect. Some of these facilitators appear to be available to a portion of the nurse leaders who are able to be part of the health policy development process, although there is a significant proportion that is excluded from the policy development process. Nurse leaders appear to want to be part of the health policy development process. There is opportunity for their participation to be enhanced in terms of involving higher numbers of nurse leaders at policy development level; and those who already occupy national positions the process must be more inclusive and open to ideas, suggestions and input from nurse leaders who are, and will be, part of the process. However, this can only occur if barriers to participation in the policy development process are overcome.

### Implications for practice, education and research

#### Nursing practice

The health policy development process needs to be pluralistic and inclusive of all nurse leaders practicing in positions related to policy development. Additionally, nurse Leaders need to proactively reexamine their own roles with regards to health policy development and strive to formalize these roles with job descriptions that include participation in health policy development. Furthermore, there is need for development of competencies by leaders that would foster inclusion in the process. Nurse leaders, through their professional organizations and their positions, need to lobby and create an enabling environment that will engender greater involvement of nurses in this arena, proactively.

### Nursing education

Nursing education as a whole needs to engender health policy development as a core area of nursing practice and relate clinical practice, education, research and leadership content to broader health policy implications. The nursing curriculum needs to reflect health policy education as a significant component of the educational process. Exposure to health policy education at bachelor’s level education may influence Nurses’ interest in the field, beyond clinical nursing. Nurse educators need to take an active role in health policy development activity and provide exposure for nurses as well as being role models and mentors in this area of practice. Furthermore nursing books and literature at basic level need to reflect this vision clearly as part of the nursing role.

### Nursing research

The findings of this study could be extended in future to study a broader sample of Nurses’ and Nurse Leaders’ at micro, meso and macro levels of their practice, exploring how nurses envision their involvement in health policy development being enhanced. There is need for action research to improve Nurses’ participation in health policy development, by extending the work of other researchers like Primomo (2013) with nursing students. Furthermore, there is the need to explore barriers that did not attain high degree of consensus as these may indicate differences within the sample, and hierarchy.

### Limitations of the study

A number of study limitations need to be acknowledged. The level of consensus set for this study was high, being at 90% for the second round while for the third round, it was 70%. This threshold was intended to ensure that only critical issues were retained in the study in the second round and that in the third round, important issues were not lost. This might have led to elimination of some significant issues. Several areas where there was lack of consensus emerged, and this might constitute a limitation of this study. Lack of consensus may be related to the proportion of the expert panelists from various sectors which might have affected the retention of certain issues such as gender related ones. Perceptions of the different sub-groups might be different from the perceptions of the group as a whole. These need to be noted and considered when interpreting the research findings. The study was conducted in the three East African countries of Kenya, Tanzania and Uganda. Other countries in East Africa were omitted. Therefore, the findings are applicable only to the countries where the study was conducted. A disadvantage of the Delphi survey method is that expert panelists may change their minds during the course of the study. This might have been the case in this study where a number of barriers were cited by the expert panelists in the first round; however during the iterative rounds several barriers were eliminated. The sample was selected purposively as per the researcher’s knowledge of the contribution that the expert panelists could make to the study. This may have resulted in some relevant nurse leaders being excluded. And as Polit and Beck [45] caution, it is likely that a segment of the population will be systematically underrepresented; therefore, interpretation of the findings must be made with caution.

## Conclusion

The study is unique in the African context and particularly in the East African context that includes Kenya, Uganda and Tanzania, where little is known about the factors that are facilitators or barriers to national nurse leaders’ participation in health policy development because the phenomenon appears to be under explored. The findings of the study indicate that both facilitators and barriers to policy involvement exist. The former include: being involved in health policy development, having knowledge and skills, being supported, enhancing the image of nursing and enabling structures and processes. The latter include: lack of involvement, the poor image of nursing and structures and processes which exclude them. Nurse leaders have a key role in mentoring, supporting and developing future nurse policy makers. There appears to be a window of opportunity as there are more facilitators than barriers; therefore, there is presently greater prospect for enhancing their participation in health policy development. The findings accrued from this study have aided in the development of an empowerment model for nurse leaders’ participation in health policy development.

### What is already known on this topic

•Most of the research contribution is from the more developed countries

•Nurses are at various levels along the continuum of political activity to influence health policy in different countries

•Health policies influence nursing positively or negatively

•There are factors that facilitate or deter nurses ability to participate in health policy

### What this study adds

•This study draws attention to the developing world and in particular the EA region, which has been largely unexplored in the area of, factors that are facilitators or barriers to national nurse leaders’ participation in health policy development

•Nurse leaders have opportunity to enhance their participation in health policy as there are number of facilitators in this regards that include: being involved; being knowledgeable and skilled, being supported; positive image of nursing; enabling structures and available resources

•Nurse leaders do have to overcome significant barriers that deter their participation that include: lack of involvement; negative image of nursing; lack of enabling structures and lack of resources

•Facilitators and barriers are largely interlinked

## Competing interest

The author declares that there are no competing interests.

## Author’s contributions

The author was involved in conception of the study; protocol development; data collection and analysis and writing the manuscript.

## Pre-publication history

The pre-publication history for this paper can be accessed here:

http://www.biomedcentral.com/1472-6955/13/20/prepub
